# Effects of Composite Coatings Functionalized with Material Additives Applied on Textile Materials for Cut Resistant Protective Gloves

**DOI:** 10.3390/ma14226876

**Published:** 2021-11-15

**Authors:** Paulina Kropidłowska, Magdalena Jurczyk-Kowalska, Emilia Irzmańska, Tomasz Płociński, Radosław Laskowski

**Affiliations:** 1 Department of Personal Protective Equipment, Central Institute for Labour Protection—National Research Institute, 48 Wierzbowa, 90-133 Lodz, Poland; pakro@ciop.lodz.pl; 2 Faculty of Material Science and Engineering, Warsaw University of Technology, 141 Woloska, 02-507 Warsaw, Poland; magdalena.jurczyk@pw.edu.pl (M.J.-K.); tomasz.plocinski@pw.edu.pl (T.P.); 3 THOREX Sp. J., 228 Kilińskiego, 93-124 Lodz, Poland; rlaskowski@thorex.com.pl

**Keywords:** protective gloves, cut resistance, functionalization of textile materials, mineral additives

## Abstract

The objective of the present work was to evaluate the effects of different types of particles added to a polymer paste applied onto a textile carrier on the cut resistance of the resulting material. Knitted aramid textile samples were coated in laboratory conditions using a polymer paste that was functionalized with 12 types of reinforcing particles of different chemical compositions and size fractions. Cut resistance was tested in accordance with the standard EN ISO 13997:1999 and the results were subjected to statistical analysis. The effects of additive particles on the microstructure of the polymeric layer were assessed by means of scanning electron microscopy. The type and size of the particles affected the cut resistance of the functionalized knitted fabric. They were also found to change the morphology of the porous structure. Composite coatings containing the smallest additive particles exhibited the best cut resistance properties.

## 1. Introduction

Workers’ hands are at the highest risk of being affected by harmful factors in the work environment and upper limb injury is the most widespread type of workplace accident occurring during the performance of manual tasks. The risk of hand injury can be mitigated by means of protective gloves, which should be selected depending on the type of hazard(s) present and the kind of occupational task(s) performed [1,2].

For safety gloves to effectively prevent mechanical injury, they need to meet a number of protective requirements imposed by Regulation (EU) 2016/425 of the European Parliament and of the Council of 9 March 2016 on personal protective equipment and repealing Council Directive 89/686/EEC (2016). In terms of mechanical hazards, a particularly salient protective parameter is cut resistance. The basic standard delineating requirements for gloves protecting against mechanical factors, including cuts, is EN 388:2016 + A1:2018 [3]. Materials claimed to exhibit high cut resistance are tested pursuant to EN ISO 13997:1999 [4], which produces more consistent results [5,6] by reducing the impact of blade blunting on the test procedure [6,7]. Cut resistance is defined as the ability of the material to withstand cutting with a blade and can be determined by means of a variety of testing and assessment methods [6]. Samples in the present study were tested for cut resistance in accordance with the standard method used for evaluating protective gloves, i.e., that specified in EN ISO 13997. It should be noted that prior to 2016 that method was not widely used for the evaluation of protective gloves. It was not until the major update of EN 388, the main standard concerning gloves protecting against mechanical risks, that the EN ISO 13997 method was acknowledged as more reliable and leading to more consistent results [5,6]. According to the literature, the sharpness of the test blade substantially deteriorates in the course of testing materials exhibiting high cut resistance [7].

To ensure optimum hand protection, gloves exhibiting high cut resistance can be made from para-aramid, polyethylene, and core-spun yarns, as well as glass fibers [5,8,9,10]. Increasing user expectations and requirements have driven research on the development of new technological solutions [11,12]. State-of-the-art textile products should be not only innovative and comfortable to wear, but they should also be designed to offer the highest possible level of safety. Innovative properties and unique functional characteristics can be imparted to textile materials by, e.g., physical and chemical surface modifications and nanoparticle deposition [13,14,15,16,17]. The ongoing technological development has fueled user expectations in terms of the properties exhibited by textile materials, which has motivated numerous efforts to functionalize such materials with the view to improving their characteristics [18,19].

The protective properties of glove materials may be improved by continuous or spot coating [20] with polymeric materials such as poly(vinyl chloride), polyurethane, silicone, and natural or nitrile rubber [21,22,23,24]. There is scant literature dealing with the functionalization of textile materials by modeling convex 3D structures to improve cut resistance. Among works devoted to the application of surface structures on textile materials, few have studied the impact of continuous coatings (applied in the form of pastes) on the cut resistance of protective gloves. In terms of mechanical properties, existing research on polymeric coatings has dealt with the effects of a polyurethane paste on elongation and tensile strength [25,26] and the influence of a thermoplastic polymer on stab resistance [27]. A promising direction of research on the strength properties (including cut resistance) of textile materials is the application of reinforcing additives in pastes. As a result of research efforts, structural additives such as micro- and nanofillers have found increasingly wide applications in elastomer blends [28]. For instance, silica has been added to polyurethane to increase hardness and Young modulus [29], while boron carbide has been added to epoxy resin to strengthen the mechanical properties of the resulting material [30].

Fillers alter the physical and chemical properties of polymers. The size, shape, and chemical composition of the additives often significantly affect the formation of porous structures (pore size and shape), modifying the mechanical characteristics of the resulting polymeric foams [31,32,33].

An interesting research area involves the application of mineral additives in coating pastes to reinforce knitted protective gloves, as well as the evaluation of their mechanical properties, such as cut resistance. For the purpose of assessing the feasibility of this approach, the present authors applied reinforcing additives, including mineral particles, in a polymer paste to increase the cut resistance of the end products.

The work evaluated the effects of 12 types of composite coatings on the cut resistance of spot-coated knitted glove materials. Furthermore, the type and particle size of additives were analyzed in terms of their influence on the porous structure of the coatings and implications for the cut resistance of the system.

## 2. Materials and Methods

### 2.1. Materials

The study material consisted of 13 spot-coated textile samples fabricated in laboratory conditions. The composite coatings were made using an acrylic-styrene polymer paste (Thotex Sp. J., Lodz, Poland) with particulate additives, including mineral fillers (Table 1). The reference material consisted of a textile carrier spot-coated with the polymer paste without additives.

The textile carrier was a knitted aramid fabric (S.I. ZGODA, Konstantynow Lodzki, Poland) with a surface density of 234.1 g/m^2^, a thickness of 0.23 mm and cut resistance of 6 N.

### 2.2. Sample Preparation

**Figure 1:**
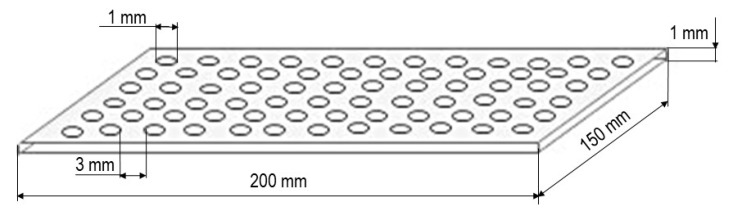
The developed geometric stencil.

**Figure 2:**
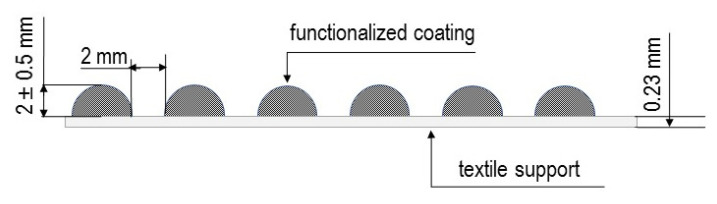
Schematic view of the samples.

The fabrication of study materials involved the following steps:

(1) Step 1: Selection of a homogeneous textile carrier—the selected carrier was a commercially available knitted aramid fabric designed for use in commercial gloves. Textile carrier samples measuring 210 × 297 mm were positioned horizontally.
(2) Step 2: Selection of a coating method—coating was carried out by screen printing using a geometric stencil (Figure 1). Convex structures were made using an acrylic-styrene polymer with a foaming agent.
(3) Step 3: Preparation of composite paste—reinforcing additives were added to the polymer paste at 20% *w*/*w* (the paste to reinforcing agent ratio was 4:1—one sample was prepared using 25 g of the polymer paste and 6.25 g of an additive).
(4) Step 4: Functionalization of the textile carrier—a metal geometric stencil was placed on a horizontal aramid fabric (Figure 1) and pressed with a force of 50 N. A composite paste was transferred onto the textile carrier using an elastic spatula, and then the stencil was removed.
(5) Step 5: Fixing of the coating elements—the samples were heated in a circulating air laboratory oven (Zalmed, Lublin, Poland) at 140 °C for 10 min. Subsequently, the samples were removed from the oven and the homogeneity of the coating elements was evaluated. A schematic view of the resulting samples is given in Figure 2.

### 2.3. Testing Methods

The coated fabrics were tested for cut resistance, which is an essential parameter of protective glove materials. Furthermore, microscopic examinations were conducted to evaluate the effects of the applied reinforcing particulate additives on the morphology of the coating and the cut resistance of the material.

#### 2.3.1. Morphology Evaluation

The microstructure of cross-sections taken from the sample was examined using a SU-8000 scanning electron microscope (Hitachi, Hitachi-shi, Japan) in the magnification range of 30–500× at an electron acceleration voltage of 5 kV. The sample cross-sections were attached to the microscope table using a conductive tape and sputter-coated with gold at 10 kV for 10 min using a Gatan high vacuum sputter coater (Pleasanton, CA, USA).

#### 2.3.2. Cut Resistance Testing

Cut resistance was tested with a straight blade using a tonodynamometer (Kontech, Lodz, Poland) according to the standard EN ISO 13997:1999 [4]. The cuts were achieved in blade movements of 3 mm to 50 mm lenght when a range of forces are applied to the blade normal to the specimen surface. Blades had ground to a bevel width of (2.5 ± 0.2) mm along straight edge—this included angle of approximately 22° at the cutting edge. Blades had a cutting-edge length 70 mm and 19 mm wide and were made of stainless steel with a hardness greater than 50 HRC. The cut resistance of material is expressed as the cutting force that is required to be applied to a blade of standard sharpness to just cut through the material in a 20 mm blade stroke. At least 20 measurements are made on each sample using new blade for each cut. The cut resistance test is conducting as follow: (a) apply a selected force progressively between the specimen and the blade; (b) make trial cuts tests to establish a force resulting in a cutting stroke length between 5 mm and 50 mm; (c) repeat tests with different forces until at least 15 readings have been obtained with cutting stroke lengths distributed between 5 mm and 50 mm; (d) normalize cutting stroke length by multiplication blade sharpness correction factor and recorded stroke length; (e) use calculated force to obtain at least five cutting stroke length between 18.0 and 22.0 mm and include these results in a recalculation of the cutting force.

Testing samples were mounted on a cylinder with a radius of (38 ± 0.5) mm. During the test, a variable force ranging from 1.0 N to 200.0 N was applied to the blade. The cutting rate was (2.5 ± 0.5) cm/s. The results were interpreted using the requirements stipulated in Table 2. Prior to testing, the samples were acclimatized at (23 ± 2) °C and a relative humidity of (50 ± 5)% for 24 h.

#### 2.3.3. Statistical Analysis

Study results were subjected to statistical analysis implemented in SPSS Statistics 25.0. Analysis of variance (ANOVA) was carried out with a posteriori bootstrapping (1000 replicates). The Tukey test was used for post-hoc comparisons. The objective of the analysis was to compare the cut resistances of the studied textile carrier functionalized with composite coatings containing various reinforcing additives.

## 3. Results

### 3.1. Morphology

SEM images of of the particles are presented in Table 1. In this study, 4 groups of fillers were used to modify the polymer paste. First group of particles—Al_2_O_3_, have multiple-walled and irregular shape (particles of additives No. 1–4). Particles of additives No. 2 and 4 have sharp edges. The second group of particles—SiC (additive No. 5–7) are characterized by a variety of morphologies and sizes. The third group of particles—SiO2 CaO (additive No. 8, 9) have a similar size. Particles 9 have sharp edges. The fourth group—glass particles (additive No. 10, 11) have an irregular shape and sharp edges. The particle morphology of additive No. 12 is typical of glass beads.

**Figure 3:**
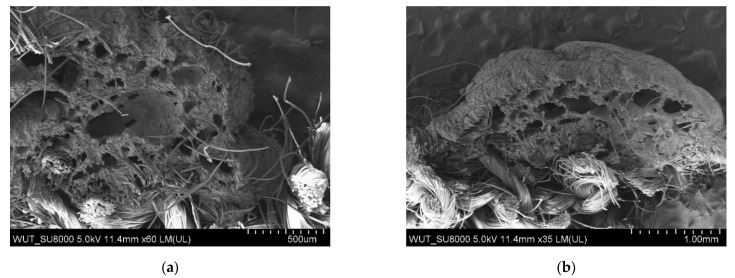
SEM images of the reference coating magnification (**a**) ×60 (**b**) ×35.

**Figure 4:**
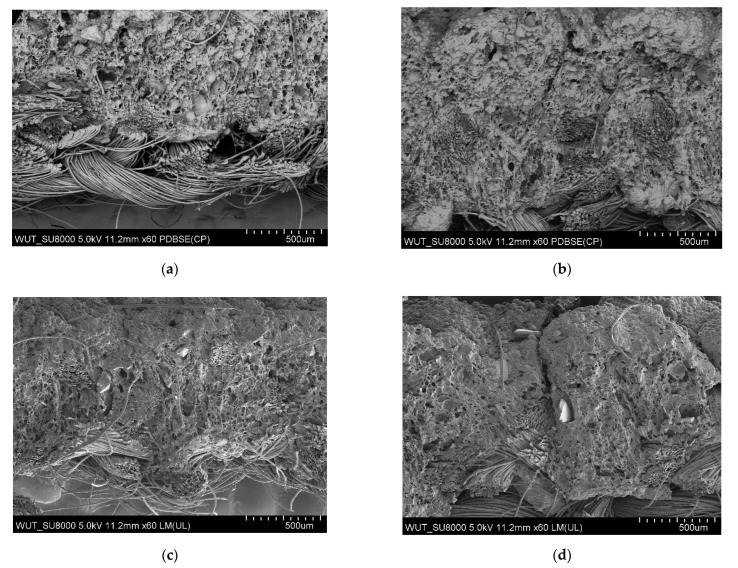
(**a**) SEM image of coating incorporating Al_2_O_3_ with a particle size of 710–750 μm (additive 1). (**b**) SEM image of coating incorporating Al_2_O_3_ with a particle size of 180–250 μm (additive 2). (**c**) SEM image of coating incorporating Al_2_O_3_ with a particle size of 100–500 μm (additive 3). (**d**) SEM image of coating incorporating Al_2_O_3_ with a particle size of 50–56 μm (additive 4).

**Figure 5:**
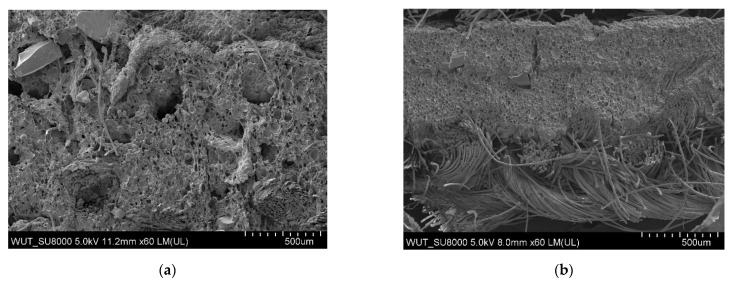

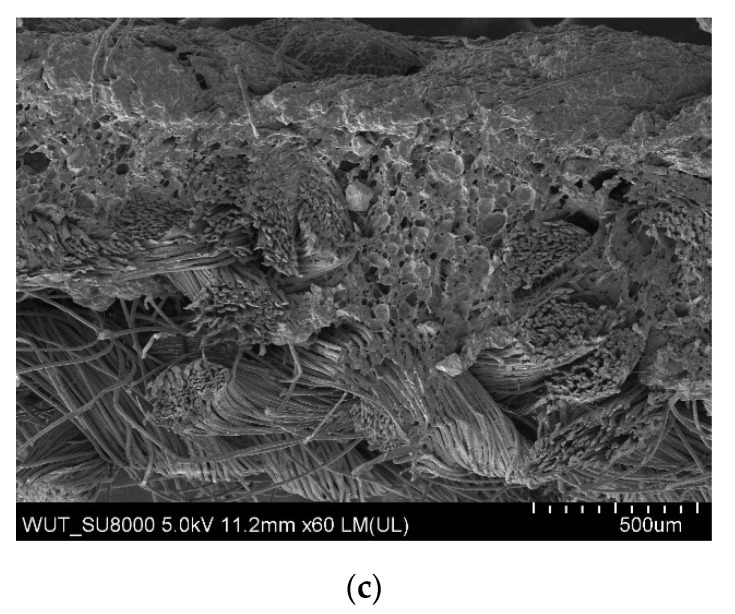
(**a**) SEM image of coating incorporating SiC with a particle size of 100–450 μm (additive 5). (**b**) SEM image of coating incorporating SiC with a particle size of 50–450 μm (additive 6). (**c**) SEM image of coating incorporating SiC with a particle size of 53–75 μm (additive 7).

**Figure 6:**
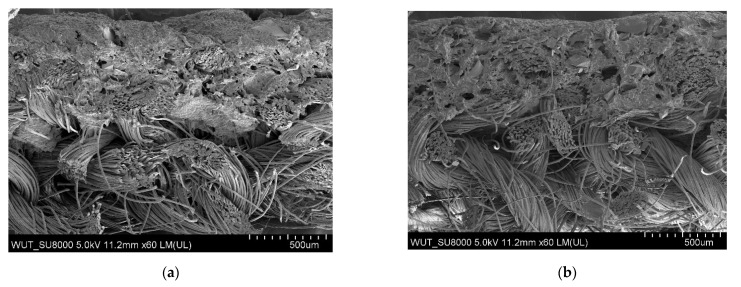
(**a**) SEM image of coating incorporating SiO_2_ + CaO with a particle size of 50–200 μm (additive 8). (**b**) SEM image of coating incorporating SiO_2_ + CaO with a particle size of 10–250 μm (additive 9).

**Figure 7:**
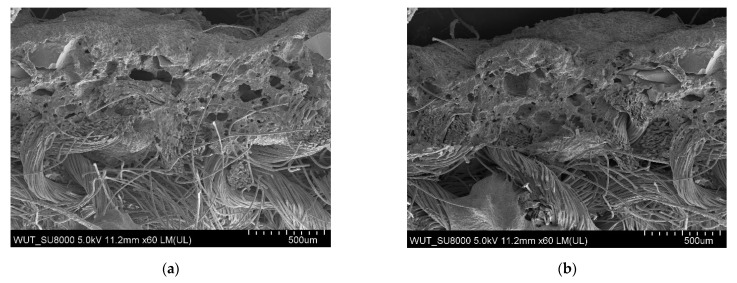

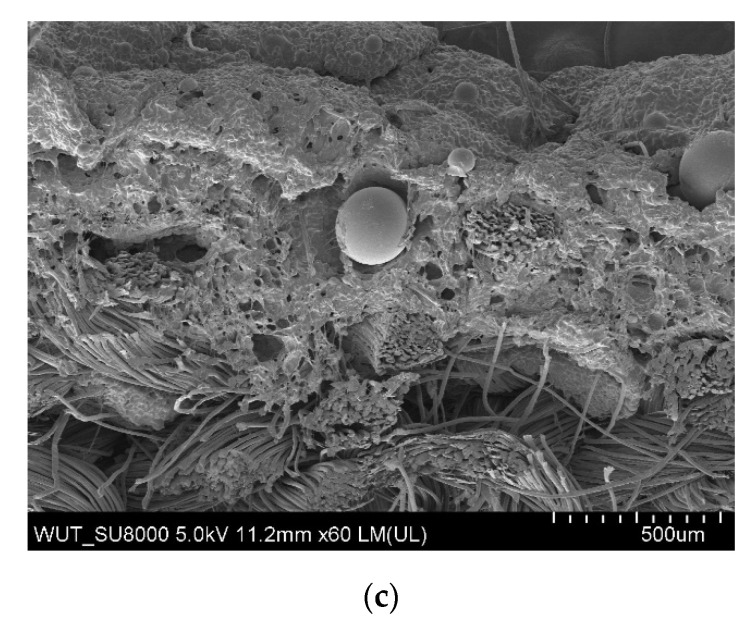
(**a**) SEM image of coating incorporating glass particles with a size of 315–500 μm (additive 10). (**b**) SEM image of coating incorporating glass particles with a size of 250–350 µm (additive 11). (**c**) SEM image of coating incorporating glass beads with a diameter of 125–630 μm (additive 12).

The largest pores were found in the polymeric layer without any reinforcing additives (Figure 3). SEM images of the examined systems are presented in Figure 4, Figure 5, Figure 6 and Figure 7. The application of all particulate additives reduced pore size in the polymer paste. The paste was modified using four groups of additives: Al_2_O_3_ (additives 1–4), SiC (additives 5–7), calcium silicate (additives 8 and 9), and glass particles (additives 10–12). Microscopic examinations have revealed that both the type and particle size of additives affected the porous structure of the polymer paste, but the identified relationships differed between the various groups of additives. In the case of Al_2_O_3_, the thinnest coating was obtained for a particle size of 100–500 µm (coatings were much thicker for the other Al_2_O_3_ particle sizes). Coating thickness decreased with decreasing particle size for composites incorporating SiC additives (Figure 5a–c). Coatings containing a mixture of SiO_2_ and CaO as well as those with glass additives exhibited similar thickness levels. In those cases, it was mostly particle size rather than porous microstructure that affected cut resistance.

### 3.2. Cut Resistance Results

**Figure 8:**
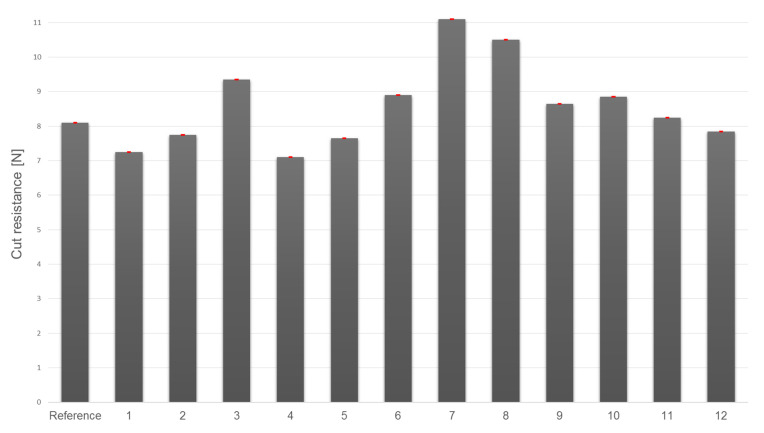
Cut resistance results.

Figure 8 presents the results of cut resistance tests for the test textile carrier functionalized with 12 composite coatings. The tests evaluated the effects of reinforcing particulate additives on the level of cut resistance offered by the materials.

The studied variants of knitted fabric functionalized with composite layers exhibited cut resistance corresponding to performance levels B and C. The highest cut resistance values were found for the variants incorporating 53–75 µm SiC particles (additive 7) and 50–200 µm SiO_2_ and CaO particles (additive 8). The cutting forces determined for those variants (11.1 N and 10.5 N, respectively) placed them at performance level C. Four variants containing 50–450 µm SiC particles (additive 6), 250–350 µm glass particles (additive 11), 315–500 µm glass particles (additive 10), as well as 10–250 µm SiO_2_ and CaO particles (additive 9) showed similar cut resistances ranging from 8.3 to 8.9 N.

Finally, five variants exhibited cut resistance below the reference sample levels; these incorporated 180–250 µm Al_2_O_3_ particles (additive 2), 50–56 µm Al_2_O_3_ particles (additive 4), 710–750 µm Al_2_O_3_ particles (additive 1), 100–450 µm SiC particles (additive 5), and glass beads (additive 12).

### 3.3. Statistical Analysis

Table 3 presents descriptive statistics for cut resistance depending on the additive used.

Table 4 presents a summary of ANOVA statistics for the effects of the studied additives on the cut resistance of the coated fabrics.

The type of additive used was found to significantly affect cut resistance. Post hoc comparisons conducted by means of the Tukey test revealed that samples 3, 7, and 8 reached significantly higher values than the reference material (coated without additives), while samples 4, 5, and 1 reached significantly lower values. Statistical analysis confirmed the significance of differences between the studied additives in terms of their effects on cut resistance.

## 4. Discussion

New and unique functional properties can be imparted to textile materials by means of physical and chemical surface modifications as well as by the incorporation of nanoparticles [13,14,15,16,17]. The proposed method of functionalizing a textile carrier with a polymer paste incorporating reinforcing additives of different chemical compositions and particle sizes improved the cut resistance of some material variants. According to literature data, cut resistance depends in particular on the type and structure of fibers [34] as well as the application of strong polymeric materials [35]. Para-aramid fibers have been reported to exhibit high cut resistance while being suitable for glove applications [7,36].

In the present study, a knitted aramid carrier was functionalized with a composite coating. Continuous and discontinuous polymeric coatings have been previously applied to improve glove properties, and especially abrasion and cut resistance [35]. Such coatings are typically made of poly(vinyl chloride) (PVC), polyurethane (PU), nitrile butadiene rubber (NBR), natural rubber (NR), and silicone rubber [20,37]. Matković et al. [25] applied a continuous polyurethane coating onto knitted fabrics to improve their mechanical properties. The application of a polyurethane paste was found to improve the elongation and tensile strength of the samples as compared to controls; the mean force needed to break them increased by 24%. Mayo et.al. [27] reported that laminated fabrics exhibited higher cut and stab resistance than non-coated materials. Gloves can also contain more than one coating layer (differing in terms of their properties), especially in situations where a single layer would fall short of the required performance levels [5]. Yang et.al. [38], who studied the effects of adding B_4_C to the coating of a woven fabric, reported the highest stab resistance for samples with multi-layer coatings.

In the present study, an acrylic-styrene paste with a foaming agent was used to form 3D structures of a specific geometry and thickness. All samples were made with one type of polymer paste incorporating different particulate reinforcing additives (Table 1), such as aluminum oxide (Al_2_O_3_), silicon carbide (SiC), as well as materials derived from quartz sand with the addition of sodium carbonate (Na_2_CO_3_) and calcium carbonate (CaCO_3_), with particle sizes ranging from 10 µm to 750 µm. Nunes et al. [29] investigated the effects of silica on the mechanical properties of an elastomeric mix, especially in terms of hardness and Young modulus. The effects were found to be strongly associated with the presence of silane groups on the surface of silica. The authors concluded that the greater the surface density of silanol groups, the stronger the elastomer.

The stab resistance of aramid composite fabrics with a thermoset coating containing SiC particles was evaluated by Rubin et al. [39]. The presence of SiC particles was found to improve stab resistance with the highest effectiveness at a concentration 20 wt.%. Microstructural investigations revealed SiC particles to be embedded in interstitial spaces. According to Rubin et al. [39], the improved stab resistance was attributable to increased friction between the fibers, which may have blunted the blade.

The greater the force needed to make a cut, the greater the cut resistance of the sample. In this work, the highest cut resistance was found for samples with the coating layer containing the SiC additive with a particle size of 53–75 µm (Figure 8); in this case the increase with respect to the reference sample (without any reinforcing additives) amounted to 37%. The second-best result (a 30% increase) was obtained for a combined application of SiO_2_ and CaO with a particle size of 50–200 µm (additive 8). The third most effective additive was Al_2_O_3_ with a particle size of 100–500 µm (additive 3), which improved cut resistance by 15% (Figure 8).

Literature reports have indicated that the cut resistance of knitted fabrics can be improved by modification with mineral additives (e.g., SiC). Research has also shown that of the essence is the size of particles added as well as their percentage share in the coatings [30,38,39]. In the present study, only a slight improvement in cut resistance (2% to 10%) was observed for four of the investigated additives, which were thus not deemed promising: SiC with a particle size of 50–450 µm (additive 6), glass with particle sizes of 250–350 µm (additive 11) and 315–500 µm (additive 10), as well as a mixture of SiO_2_ and CaO with a particle size of 10–250 µm (additive 9). Finally, statistical analysis revealed a significant increase in cut resistance for additives 3, 7, and 8 (Table 3 and Table 4).

In the present study, the application of some additives was found to decrease cut resistance by 2% to 10%; these included Al_2_O_3_ with a particle size of 50–750 µm (additives 1, 2, and 4), SiC with a particle size of 100–450 µm (additive 5), and glass beads (additive 12) (Figure 8).

In the context of these findings, it should be noted that Gent and Wang [40] showed that the energy of cutting through polymeric materials consists of two components: the energy needed to break molecular chains and the energy of viscoelastic and plastic deformation (depending on the type of material). Moreover, according to the literature cut resistance largely depends on the friction between the material and the blade. Importantly, a higher friction coefficient may either increase or decrease cut resistance, depending on the properties of the material, including its thickness and microstructure [41]. To explain the observed cases of diminished cut resistance, the present authors additionally performed microstructural examinations using scanning electron microscopy.

In the case of Al_2_O_3_ additives, the cut resistance of the composite layer was improved for a particle size of 100–500 µm and deteriorated for the remaining particle sizes (Figure 8). Microstructure examinations revealed that the coating incorporating 100–500 µm Al_2_O_3_ particles was the thinnest and penetrated between the fibers of the knitted fabric carrier, forming a compact layer (Figure 4c). In addition to the size of particles in the polymer paste, the cut resistance of the studied systems was also largely influenced by the way in which the paste penetrated into the knitted fabric structure.

In the case of the coating containing SiC, cut resistance decreased with increasing additive particle size. It was also found that the finer the additive particles, the thinner the composite layer and the better its fiber wetting properties (Figure 5a–c). As regards the SiC additives, the highest cut resistance was found for 53–75 µm particles (Figure 5c).

Both coatings incorporating a mixture of SiO_2_ and CaO particles exhibited improved cut resistance vs. the reference sample, with the smaller particles (50 µm) being more effective (Figure 6a). The last group of additives consisted of glass beads and glass particles differing in their shape and size (Figure 7a–c). While glass particles improved cut resistance (again the smaller particles turned out to be superior), the application of glass beads compromised the cut resistance of the system as compared to the reference sample (Figure 3).

## 5. Conclusions

Polymeric coatings can increase the mechanical resistance of gloves, thus enhancing their functional properties. This study presents functionalized materials designed to improve the cut resistance of protective gloves. A knitted fabric carrier was coated with a composite paste containing a wide range of additives. The greatest increases in cut resistance were found for Al_2_O_3_ with a particle size of 100–500 µm (additive 2), SiC with a particle size of 53–75 µm (additive 7) as well as SiO_2_ and CaO with a particle size of 50–200 µm (additive 8). In the case of a mixture of SiO and CaO (additive 9) and glass particles (additives 10 and 11), cut resistance increased only slightly as compared to the reference sample without additives. Analysis of the microstructure of the studied textile carrier with composite coatings revealed that layers with the lowest thickness, which penetrated into the structure of the carrier (as in the case of additives 2—thickness: 2.40 mm and 6—thickness: 2.20 mm) constituted the most effective barriers to blades in cut resistance tests. Particle size was found to affect the porous microstructure of the polymer paste and cut resistance properties. Smaller additive particles in the polymer paste more readily penetrated between the fibers of the knitted fabric, in this way preventing the process of forming porous structures and creating a more mechanically resistant layer.

## Figures and Tables

**Table 1:** Reinforcing additives.

Additive No.	SEM Images (Magnification ×50)	Chemical Composition	Physical Properties (Particle Size)
1	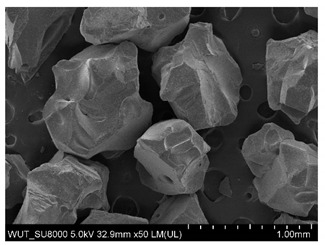	Al_2_O_3_	710–750 µm
2	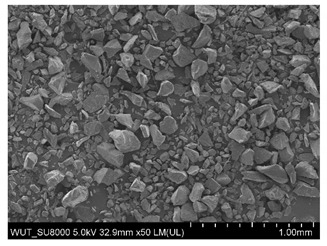	Al_2_O_3_	180–250 µm
3	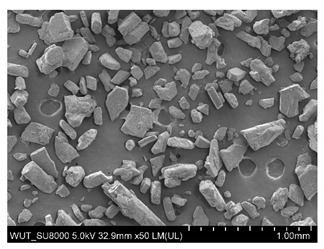	Al_2_O_3_	100–500 µm
4	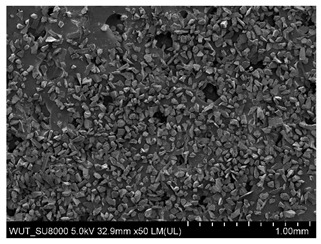	Al_2_O_3_	50–56 µm
5	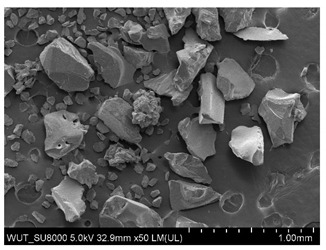	SiC	100–450 µm
6	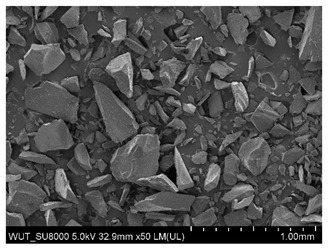	SiC	50–450 µm
7	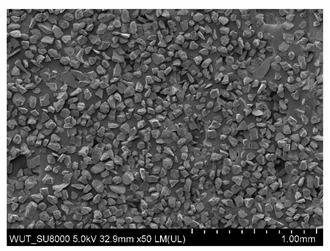	SiC	53–75 µm
8	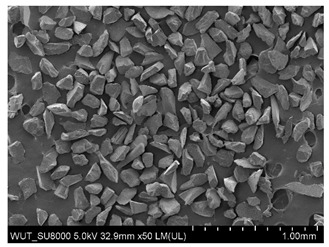	SiO_2_ CaO	50–200 µm
9	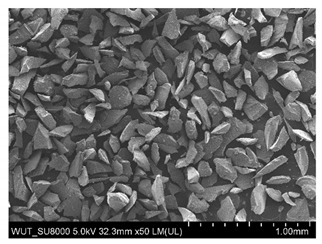	SiO_2_ CaO	10–250 µm
10	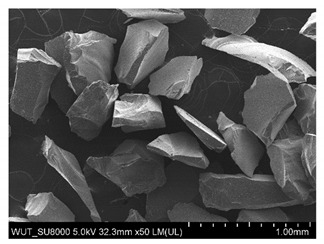	SiO_2_ > 65% Na_2_O > 14% CaO > 8.0% MgO < 4.0% Al_2_O_3_ 0.5–2.0% Fe_2_O_3_ < 0.2%	315–500 µm
11	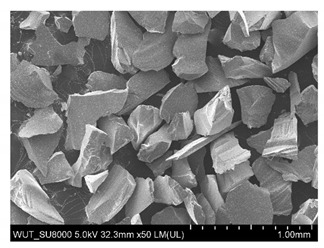	SiO_2_ > 65% Na_2_O >14% CaO > 8.0% MgO < 4.0% Al_2_O_3_ 0.5–2% Fe_2_O_3_ < 0.2%	250–350 µm
12	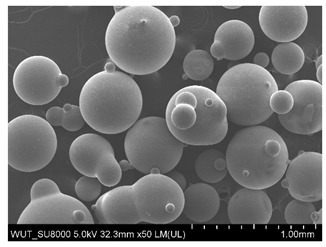	SiO_2_ 70–73% Na_2_O + K_2_O 13–15% CaO 7–11% MgO 3–5% Al_2_O_3_ 0.5–2.0% TiO_2_ ≤ 0.1%	125–630 µm

**Table 2:** Performance levels for cut resistance [3].

Performance Level	Level A	Level B	Level C	Level D	Level E	Level F
Cutting force [N]	2	5	10	15	22	30

**Table 3:** Descriptive statistics of cut resistance for the studied additives.

Parameter	Additive	N	Min	Max	M	SD
Cut resistance [N]	Reference material (without additives)	2	8.20	8.10	8.15	0.07
	1	2	7.40	7.10	7.25	0.21
	2	2	7.90	7.60	7.75	0.21
	3	2	9.50	9.20	9.35	0.21
	4	2	7.30	6.90	7.10	0.28
	5	2	7.80	7.50	7.65	0.21
	6	2	9.00	8.80	8.90	0.14
	7	2	11.20	11.00	11.10	0.14
	8	2	10.80	10.20	10.50	0.42
	9	2	8.90	8.40	8.65	0.35
	10	2	9.10	8.60	8.85	0.35
	11	2	8.40	8.10	8.25	0.21
	12	2	8.10	7.60	7.85	0.35

**Table 4:** ANOVA statistics for the effects of the studied additives on cut resistance.

Parameter	F (12, 13)	*p*	η^2^	Post-Hoc Test
				0 < 3,7,8; 0 > 4
Cut resistance				1 < 8,11,10,9; 2 < 3,6,7,8,10;
	41.05	0.001	0.97	2 > 3,4,1,8; 3 < 7,8; 4 < 6,7,8,11,10,9;
				5 < 6,7,8,10; 6 < 7,8; 6 > 1,12;
				7 > 1,11,10,9,12; 8 > 11,10,9,12

## Data Availability

Not applicable.
